# The influence of size, depth and histologic characteristics of invasive ductal breast carcinoma on thermographic properties of the breast

**DOI:** 10.17179/excli2019-1600

**Published:** 2019-07-22

**Authors:** Marko Mance, Krešimir Bulic, Anko Antabak, Milan Miloševic

**Affiliations:** 1 Department of Plastic, Reconstructive and Aesthetic Surgery, University Hospital Center Zagreb, Kišpaticeva 12, 10 000 Zagreb, Croatia; 2 Department of Pediatric Surgery, University Hospital Center Zagreb, Kišpati?eva 12, 10 000 Zagreb, Croatia; 3 University of Zagreb, School of Medicine. Mirogojska cesta 16, 10000, Zagreb

**Keywords:** invasive ductal breast carcinoma, thermography, breast cancer, estrogen receptor, progesterone receptor

## Abstract

Invasive breast carcinoma is the most common oncologic disease worldwide. The existing diagnostic methods use morphologic changes in the breast to diagnose a carcinoma when it has reached a certain size. Therefore, it is important to augment the morphologic diagnostic examinations with a new method that focuses on characteristics other than morphology such as electromagnetic changes produced by cancer. 50 adult female patients with confirmed ductal carcinoma following a core biopsy due to a suspicious breast mass were included in the study. They underwent breast thermography using a specially designed infrared camera. The data collected was statistically analyzed to determine how the presence of a tumor and its histologic characteristics influence breast thermographic properties. Twenty eight [56 %] patients in the study had an abnormal thermogram. Following statistical analysis, it was found that temperature of the diseased breast was directly correlated to tumor volume [*p*=0.009] and negatively correlated to depth of tumor [*p*=0.042]. Tumors that were ER+ and PR+ tumors produced warmer temperatures [*p*=0.017 and *p*=0.038 respectively] than tumors without these receptors. HER2 status and Ki-67 index had no statistical correlation with breast temperature. Tumor size, distance from the skin surface and receptor status cause changes in breast thermographic properties. Despite technical advances in the field of thermography, there are still contradictory results associated with thermography. Its diagnostic abilities are generally poorer than conventional methods and its use in breast cancer screening or as an adjunctive tool for diagnostic purposes is not recommended.

## Introduction

Invasive breast cancer is the most common malignancy in women around the world accounting for 25 % of all types of cancers and the incidence has more than doubled worldwide in the last 25 years (Ghoncheh et al., 2016[14]).

In East Africa, the incidence ranges from 19.4 per 100,000 individuals while in Western Europe it is about 89.7 per 100,000. Survival rates for this disease also have varying rates with percentages as high as 80 % or over in Sweden, Japan and North America. While in middle income countries it is about 60 % and in low-income countries, below 40 % (WHO, 2015[39]).

Invasive breast cancer is a heterogeneous disease with different biological profiles and various expressions of prognostic and predictive markers. These include tumor size, lymph node stage, expression of estrogen receptor (ER), progesterone receptor (PR), histologic status, overexpression of human epidermal growth factor receptor 2 (HER-2), and Ki-67 proliferation index (Rakha et al., 2014[31]).

In the developed world, invasive breast cancer screening programs are available and encouraged. Early detection of invasive breast cancer is dependant on three standardized radiologic tests; mammography (MMG), ultrasound (US) and magnetic resonance imaging (MRI) as well as tissue sampling and analysis; fine needle aspiration (FNA) and core biopsy (CB). Despite these methods of early breast cancer detection, the incidence of this disease is constantly increasing worldwide. These methods are based on the detection of morphologic changes in the breast and have several disadvantages including varying a rates of sensitivity and specificity, high costs, ionizing radiation, contact with the patient and the requirement for a highly trained radiologist (Sardanelli et al., 2011[33]).

Despite false-negative rates ranging approximately 15 % and with decreasing sensitivity in patients on hormonal replacement therapy, mammography is considered the most reliable imaging modality in use today (Chan et al., 2015[8]).

Multiple studies on early invasive breast cancer detection report an average sensitivity range for US, MMG and MR at 82-95 %, 66-95 % and 81-89 % respectively. While specificity ranges for US, MMG and MR were 66-84 %, 89-98 % and 68-90 % respectively (Tan et al., 2014[37]; Kriege et al., 2006[22]).

Due to the increasing incidence and mortality of invasive breast cancer worldwide, emphasis on earlier detection is placed. There is a need for complimentary imaging techniques that can recognize metabolic, immunological and vascular changes associated with early tumor growth.

Hippocrates first described the use of thermo-biological diagnostics in his writings around 480 B.C. He spread a mud slurry, a semi-liquid mixture, over the patient and observed for areas that would dry faster than others. He believed that this sign indicated underlying organ pathology (Adams, 1939[1]).

Medical thermography was pioneered in Germany in 1952 when Dr. Schwamm and the physicist Reeh developed a single detector infrared bolometer that was used for sequential diagnostic thermal measurement of defined regions of the human body (Schwamm and Reeh, 1953[34]). Prior to their studies, infrared imaging cameras were mostly used for military use. These early cameras were of poor resolution (thermal as well as spatial) and were costly. There was also a lack of computer hardware and software advanced enough for these early images to be accurate enough to be used for medical diagnostic purposes.

Since the 1980's, technology thought to be suitable for medical purposes has been available and used worldwide, even though these early infrared imaging devices were of poor resolution, stability, reproducibility and exact measurement (Amalu, 2002[2]). With major advancements in microelectronic, imaging and computer development in the past 30 years, modern infrared cameras have higher resolutions and better stability, reproducibility and sensitivity (Berz and Sauer, 2007[5]).

Thermography is a non-invasive, imaging modality devoid of ionizing radiation, that measures the infrared (heat) radiation released by the body. The underlying principle by which thermography detects pre-cancerous growths and cancerous tumors is based on the theory of neoangiogenesis and resultant hypervascularity, necessary to maintain the increased metabolism of cellular growth and multiplication. Therefore, more energy in the form of electromagnetic radiation or heat is released by the tumor compared to normal surrounding tissues.

To date, the scientific community does not support thermography as a feasible method for invasive ductal breast cancer screening (Brkljačić et al., 2013[7]). Recently, advanced cameras and software have sparked a renewed interest of thermal imaging for breast cancer. The objective of this paper is to evaluate three specific parameters of invasive ductal breast carcinoma; histologic characteristics, tumor size and tumor distance from the skin and how they influence breast surface temperature.

## Materials and Methods

The research was conducted between October 2017 and August 2018. We enrolled 50 adult female patients with confirmed ductal carcinoma following a core biopsy due to a suspicious breast mass and all of them underwent a theomorphic examination using a specially designed thermocamera (TrueIR Thermal Imager, Model U5855A, Developer: Keysight Technologies, 11900 Penang Malaysis, December 2015). Patients with invasive ductal breast carcinoma were consented and included in the study, all gave written consent. Patients who had previous breast surgery of any kind, radiation therapy, inflammatory or infections of the breast, diseases of the thorax, previous chemotherapy, were pregnant, multifocal and multicentric tumors, were febrile or had a difference of breast size >1 were excluded from this study. The research was approved by the hospital's ethics committee.

**Figure 1:**
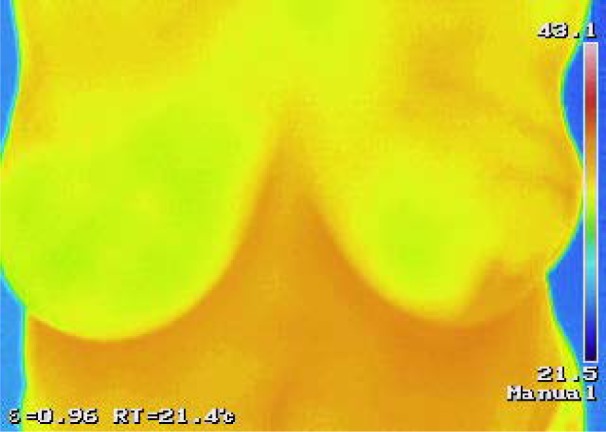
An abnormal thermogram of a female patient with invasive ductal carcinoma in her left breast. This figure clearly shows the increased temperature of the lateral left quadrant where her cancer was located.

A thermogram was taken prior to biopsy using the standardized protocol for thermographic imaging, with one photo taken from the front at distance of 1 meter, patients' hands above her head and using the same room at same atmospheric conditions. The data collected included average, maximal and minimal breast temperatures of the diseased and disease-free breast. Histologic characteristics were determined by a patho-histologic analysis following a biopsy. A thermogram was considered positive if the maximum and average breast surface temperature difference between the breasts was more than 0.5 °C (Figure 1(Fig. 1)).

This data was statistically analyzed and compared to determine if the presence of a tumor and histologic characteristics influence breast thermographic properties.

## Results

In total, 50 patients with invasive ductal breast carcinoma were enrolled in this study (age range, 34-90 years; mean age, 60.3 years). Table 1(Tab. 1) summarizes the clinical data of the patients. Of them, 28 (56 %) patients had an abnormal thermogram. It was found that the tumor area ranged between 24 mm^2 ^- 2862.00 mm^2 ^(average 314.41 mm^2^) and depth of tumor ranged between 1 mm-40 mm (average 10.93 mm). 42 patients (85.7 %) had ER positive tumors, 40 (81.6 %) had PR positive tumors and 8 (16.3 %) had HER2 positive tumors. Ki-67 index values ranged between 5 %-80 % (average 24.6 %).

Following statistical analysis, it was found that temperature of the diseased breast was directly correlated to tumor volume (*p*=0.009) and negatively correlated to depth of tumor (*p*=0.042).

When histologic characteristics were considered, breasts with ER+ and PR+ tumors were generally warmer (*p*=0.017 and *p*=0.038 respectively) than tumors without these receptors. HER2 status and Ki-67 index had no statistical correlation with breast temperature.

## Discussion

Breast cancer can be found in any part of breast tissue and there have been more than 20 types of cancer identified. The most common is ductal carcinoma which originates from the ductal epithelium (Sharma et al., 2010[35]). It is the most commonly diagnosed cancer in females and the leading cause of cancer death in this population (Bray et al., 2018[6]). Despite advances in early diagnosis and treatment, this disease continues to be a major global cause of morbidity and mortality.

There have been a variety of diagnostic imaging modalities developed to assist the physician which are aimed to improve the sensitivity and specificity of breast cancer detection. Mammography is considered the gold standard for screening purposes, with US and MRI widely used to assist or complement it. It is important to be aware that no single imaging modality is suffice to identify and characterize all possible breast abnormalities. Therefore, a combined diagnostic approach is still required (Kandlikar et al., 2017[20]).

When all three modalities are compared, multiple studies on early invasive breast cancer detection report an average sensitivity range for ultrasound, mammography and MR at 82-95 %, 66-95 % and 81-89 % respectively. While specificity ranges for ultrasound, mammography and MR were 66-84 %, 89-98 % and 68-90 % respectively (Tan et al., 2014[37]; Kriege et al., 2006[22]).

Despite the established benefits of MMG, US and MRI several disadvantages still exist including varying rates of sensitivity and specificity, high costs, ionizing radiation with considerable health risks and the need for experienced physicians to adequately read the radiologic results. Available diagnostic modalities recognize only morphologic changes in the breast and will detect a tumor when it has reached a certain size. Therefore, there is a need to complement these methods and along with morphologic changes, research other tumor properties, such as electromagnetic characteristics, which is the basis for thermography.

In 1956, Lawson reported the possible use of surface temperature measurements for breast cancer detection (Lawson, 1956[23]). Lawson and Chughtai in 1963 measured surface temperature of the region surrounding a breast tumor and found that it was about 2 ºC higher than the surface temperature of the same region on the contralateral healthy breast; a finding similarly found by Davison et al. (1972[9]). In 1982 the American Food and Drug Administration, based on the preliminary results by Gautherie that showed a relationship between breast surface temperature profile and the presence of a malignant tumor, approved thermography as an adjunct tool to mammography for breast cancer detection (Gautherie, 1980[12]).

At present, there are still mixed opinions on the usefulness of thermography as a screening or diagnostic tool for breast cancer, with certain authors in favor of the modality (Köşüş et al., 2010[21]), while others argue against it (Vreugdenburg et al., 2013[38]). This is mostly due to the inconsistent specificity and sensitivity values observed by various authors.

In one such study, a systematic search of 7 biomedical databases was conducted by Vreugdenburg et al. and noted variations in sensitivity [0.25- 0.97] and specificity [0.12- 0.85] of digital thermography. They therefore concluded that there is insufficient evidence to recommend thermography for use in breast cancer screening (Vreugdenburg et al., 2013[38]). Sajadi et al. (2013[32]) had a similar conclusion and claims that thermography does not show any acceptable diagnostic value in comparison with other diagnostic modalities. Parisky et al. (2003[29]), in their multicentric study of 769 patients, used digital thermography to image their patients prior to breast biopsy. 875 biopsies were taken and they found that the sensitivity was 97 %, and specificity only 14 %, while Aora et al. (2008[4]) observed sensitivity and specificity values of 97 % and 44 % respectively with a 82 % negative predictive value. In another paper, Wishart et al. (2010[40]) found thermography to be effective in women younger than 50 years with a high sensitivity of 78 % and specificity of 75 %. It was found that a reduced vascularity existed in the breasts of older women, especially in the group over 70 years of age. They therefore conclude that this may be the reason why thermography, in this group of patients gives a poor and unreliable result.

In addition, Omranipour et al. (2016[27]) compared the sensitivity and specificity of mammography with thermography (80.5 %, 73.3 % and 81.6 %, 57.8 % respectively). In a similar study Prasad et al. (2016[30]) found that breast malignancy was accurately detected in 60 patients (92.31 %) using thermography and 62 out of 65 patients (95.38 %) using MMG. In the latter, it was found that thermography was able to detect a malignancy in all 3 cases in which conventional mammography did not (Prasad et al., 2016[30]).

However when Yao et al. studied the effect of tumor size on thermographic characteristics, they found that infrared thermography was superior in sensitivity and specificity than mammography and ultrasound in lesions less than 2 cm in diameter. They also concluded that mammography only has a better diagnostic accuracy in lesions with a diameter larger than 2 cm (Yao et al., 2014[41]).

The results of this study suggest that size, depth and histologic characteristics of invasive ductal breast carcinoma have an influence on thermographic characteristics of the breast. There exists a variety of possbile explanations for these findings, many of which have been covered in the vast array of literature on breast thermography.

The mechanisms underlying changes of breast surface temperatures between diseased and disease-free breasts is attributed to two distinct factors; tumor induced increased nitric oxide production with resulting vasodilatation (increased metabolic activity) and neo-angiogenesis in breast tumors (increased vascularity). In their paper on pre-invasive breast cancer Guidi and Schnitt (1996[15]) reported that there is a seven times greater risk of developing breast cancer in women who have a larger number of breast micro vessels compared with women with normal micro vessel density (Guidi and Schnitt, 1996[15]). Gamagami (1996[11]) observerd the use of mammography and IR thermography for breast cancer detection and found that hypervascularity with increased local temperatures are found in up to 86 % of non-palpable breast carcinomas. The author also noted non-palpable cancers were undetected by mammography in 15 % of patients, but were recognized using IR thermography. Some authors claim that thermography can detect pathological changes 8-10 years before a mammogram can detect a mass in the body of the patient (Mambou et al., 2018[25]). Similar results were found by Gautherie and Gross who conducted a clinical study on nearly 58,000 patients and observed that healthy patients with abnormal thermograms are at a higher risk of developing cancer (Gautherie and Gros, 1980[13]).

In their research Faustino-Rocha et al. (2013[10]) induced mammary tumors in rats and using thermography, found that tumor volume was significantly correlated to maximal tumor temperature and thermal amplitude. Few authors found similar results where there is a direct correlation between the tumor size and an abnormal thermogram (Head and Elliott, 1997[16]). It is important to note that the dispersion of heat by a tumor to the skin surface is not only dependant on tumor size, but distance from the skin as well.

As in our research, tumor size and location is most important variable that affects breast surface temperature. Hu et al. (2004[18]) found that the difference of skin surface temperature when comparing breasts without and with cancer was 1.72 °C and 0.1 °C for tumors located at 2 cm and 5 cm in depth respectively. In addition, Amria et al. (2016[3]) describe that surface temperature increases between 0.2-1.2 °C for cancers between 10-30 mm in diameter which were located less than 20 mm from the surface. They found that for tumors at 1 cm from the surface and a change in diameter from 1 to 3 cm had an increase in surface temperature by 0.2 °C (Amria et al., 2016[3]). The correlation that tumor depth is the dominant factor influencing the surface temperature has been described by other authors as well (Kandlikar et al., 2017[20]). Similar conclusions were made by Jiang et al. (2008[19]) who showed surface temperature distribution is more dependant of tumor depth rather than tumor diameter. They found that tumors 2 cm or less in depth led to an increase in temperature difference between healthy and cancer affected breast by about 1.5 °C. On the other hand, they also found that tumors between 1 cm and 3 cm that were located at 2 cm or less from the skin surface changed the comparative temperature difference by about 0.1 °C. For tumors deeper than 2 cm, an insignificant surface temperature difference between normal and unhealthy breast was found.

In another study it was found that tumors located near the surface (depth range 5 mm to 18 mm) produced higher skin temperature areas that gradually increased with decreasing tumor depth and increasing tumor diameter. They also observed that tumor depths of 36 mm to 49.5 mm produced a skin surface temperature with a cold area around the tumor. The authors saw that this colder area increased as the depth of the tumor decreased and as the size increased and concluded that this phenomenon can be explained as a cooling effect due to higher blood flow and increase heat dissipation within the tumor region (Osman and Afify, 1988[28]).

The authors Ng and Sudharsan observed a warmer area on the skin surface directly above the tumor location. They found that a ratio between the tumor diameter and depth of 1:3 is a limit for producing a change in skin surface temperature. They concluded this based on their findings that a 1 cm tumor diameter and a depth of 3 cm from the surface produced a minimally noticable thermographic change (Ng and Sudharsan, 2001[26]).

There is significant data in liturature to conclude that tumor depth and diameter create significant changes on the skin surface temperature. However, as in our findings, the majority of authors have concluded that at certain tumor depths, the thermal signature is comparable to that of a healthy breast while tumors closer to the skin surface have maximum temperature differences in the range of 0.6 and 1.5 °C between healthy and diseased breasts.

In a paper published by Sterns et al. (1996[36]) a significant correlation between an abnormal breast thermogram and age, cancer stage, lymph nodal status, size, grade, and estrogen receptor status was found; but they concluded that thermography is not an independent prognostic indicator. Head et al. (1993[17]) found that tumor size and the expression of the proliferation-associated tumor antigen Ki-67 was associated with an abnormal thermogram. Unlike in our study, they found claim that neither receptor status (progesterone or estrogen) showed any clear relationship to the thermographic results. However, different results were observed by Zore et al. (2015[42]). They found that tumor size had no influence on the increased temperature. Rather, they claim that increased diseased breast temperature was more dependent on immunohistochemistry phenotypes of the tumor than any other factors. They found an association between high temperature increases in affected breast with HER-2 positive, PR negative and high Ki-67 valued tumors (Zore et al., 2015[42]).

## Conclusion

This study shows that tumor size, distance from the skin surface and receptor status causes changes in breast thermographic properties. Despite technical advances in the field of thermography, there are still a wide range of conflicting results associated with this technology. Its diagnostic abilities are generally less than those of US, MMG and MRI and there is a lack of sufficient evidence which would support breast thermography as a screening method or adjunctive tool for early breast cancer detection. Further research with a large sample group that concurrently analyzes how tumor size, depth and histologic characteristics influence thermographic properties is required.

## Conflict of interest

All of the authors claim to the best of their knowledge that no conflict of interest exists.

## Figures and Tables

**Table 1:**
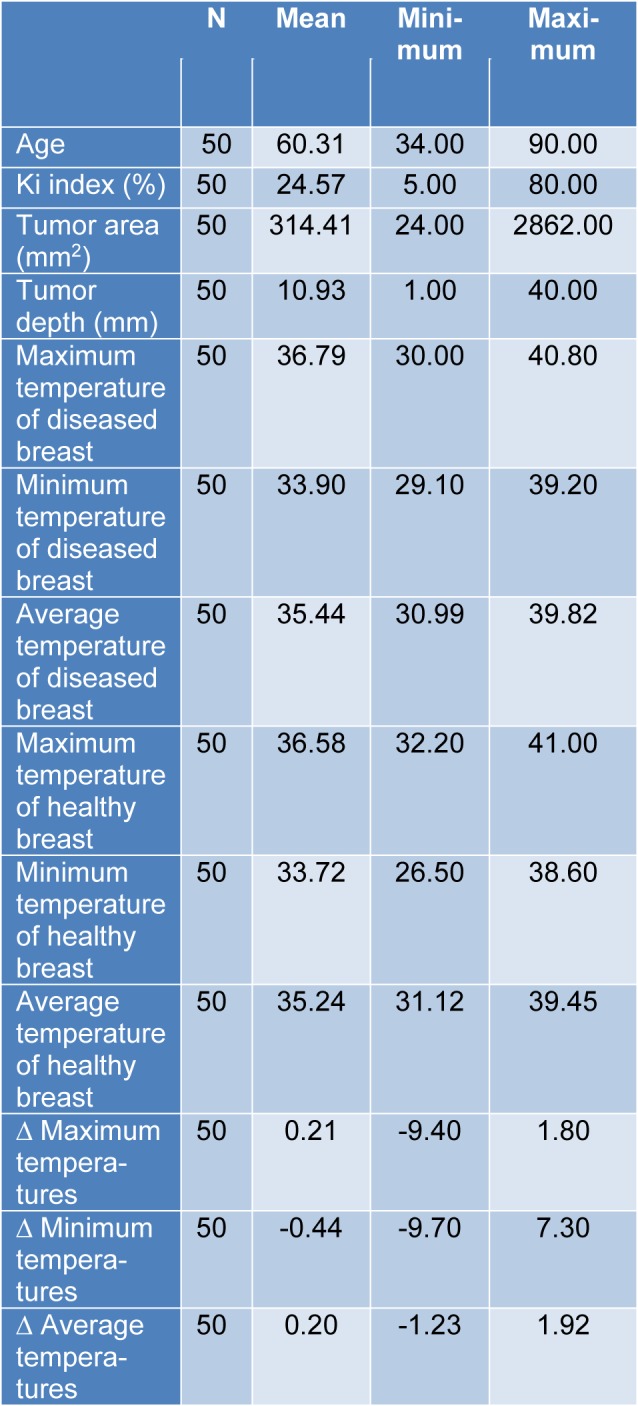
Clinical data of the patients
